# Does Oblique Effect Affect SSVEP-Based Visual Acuity Assessment?

**DOI:** 10.3389/fnins.2021.784888

**Published:** 2022-01-14

**Authors:** Xiaowei Zheng, Guanghua Xu, Yuhui Du, Hui Li, Chengcheng Han, Peiyuan Tian, Zejin Li, Chenghang Du, Wenqiang Yan, Sicong Zhang

**Affiliations:** ^1^School of Mechanical Engineering, Xi’an Jiaotong University, Xi’an, China; ^2^State Key Laboratory for Manufacturing Systems Engineering, Xi’an Jiaotong University, Xi’an, China

**Keywords:** visual acuity, steady-state visual evoked potential (SSVEP), oblique effect, stimulus orientation, spatial frequency

## Abstract

This study aimed to explore whether there was an effect on steady-state visual evoked potential (SSVEP) visual acuity assessment from the oblique effect or the stimulus orientation. SSVEPs were induced by seven visual stimuli, e.g., the reversal sinusoidal gratings with horizontal, two oblique, and vertical orientations, reversal checkerboards with vertical and oblique orientations, and oscillating expansion-contraction concentric-rings, at six spatial frequency steps. Ten subjects participated in the experiment. Subsequently, a threshold estimation criterion was used to determine the objective SSVEP visual acuity corresponding to each visual stimulus. Taking the SSVEP amplitude and signal-to-noise-ratio (SNR) of the fundamental reversal frequency as signal characteristics, both the SSVEP amplitude and SNR induced by the reversal sinusoidal gratings at 3.0 cpd among four stimulus orientations had no significant difference, and the same finding was also shown in the checkerboards between vertical and oblique orientation. In addition, the SSVEP visual acuity obtained by the threshold estimation criterion for all seven visual stimuli showed no significant difference. This study demonstrated that the SSVEPs induced by all these seven visual stimuli had a similarly good performance in evaluating visual acuity, and the oblique effect or the stimulus orientation had little effect on SSVEP response as well as the SSVEP visual acuity.

## Introduction

Visual acuity, a measure of the spatial resolution of the visual processing system, is one of the most essential parameters for testing visual ability, mainly carried out with subjective tests such as letter charts (e.g., Sloan letters or Snellen letters) (Bailey and Lovie, 1976; Ricci et al., 2009), and partially automated methods, such as the Freiburg Visual Acuity and Contrast Test (FrACT) (Bach, 1996, 2007). These methods require the subjects to have sufficient intelligence to comply with the test process and do not suit the case where subjects are unable to cooperate (e.g., preverbal or infantile children, patients with functional vision problems, the mentally disabled, and malingerers) (Incesu and Sobaci, 2011; Strasser et al., 2019; Zheng et al., 2020a).

Electroencephalography (EEG), e.g., visual evoked potentials (VEPs), have been used as an alternative method to estimate visual acuity objectively for over 40 years (Regan, 1973; Hamilton et al., 2021a,b). By varying the spatial frequency of the visual stimuli, visual acuity can be measured by establishing the mathematical model between spatial frequency and VEP signals. In general, the visual stimuli in VEP visual acuity can be checkerboards, gratings, and a novel motion paradigm of oscillating expansion-contraction concentric-rings (Zheng et al., 2019, 2020a), among which the most used stimulus patterns are sinusoidal gratings and checkerboards (Zheng et al., 2020c). Besides, the stimulus orientation of gratings is usually vertical or horizontal (Zheng et al., 2020c).

The phenomenon that visual perceptual performance is worse in response to oblique contours than to vertical or horizontal ones is called the oblique effect (Appelle, 1972; Furmanski and Engel, 2000; Li et al., 2003). Previous studies have found that VEPs are affected by stimulus orientation (Nelson et al., 1984; Moskowitz and Sokol, 1985), and its influence also shows the difference to visual stimuli with various spatial frequencies (Arakawa et al., 2000). Since the visual acuity threshold determination criterion is based on the relationship between VEP amplitude and spatial frequency (Zheng et al., 2020c; Hamilton et al., 2021b), the changes of VEP amplitude to visual stimuli with various spatial frequencies may have a direct impact on the VEP visual acuity threshold. Although VEP provides an alternative visual acuity assessment method for preverbal or infantile children and mentally disabled patients, their intelligence may not be sufficient to qualify the test process. Subsequently, their head may turn or move slightly during the test, causing the obliquity of the visual stimuli to their eyes (Moskowitz and Sokol, 1985). Hence, it is worth exploring the effect of the obliquity of the visual stimuli on VEP visual acuity. However, to date, little is known about the effect of oblique effect on VEP visual acuity assessment.

Therefore, in this study, we aimed to analyze the effect of oblique effect on visual acuity assessment using steady-state VEPs (SSVEPs) (Zheng et al., 2020a). SSVEPs were induced by reversal sinusoidal gratings with horizontal, two oblique, and vertical orientations, reversal checkerboards with vertical and oblique orientations, and oscillating expansion-contraction concentric-rings. By establishing the relationship between SSVEP amplitude and spatial frequency, SSVEP visual acuity can be obtained by threshold estimation criterion of linear extrapolation (Yadav et al., 2009; Hamilton et al., 2021a).

## Materials and Methods

### Participants

Ten healthy volunteers (three females, ages 22–27 years) with normal or corrected normal visual acuity were recruited from Xi’an Jiaotong University. The subjective visual acuity was evaluated by FrACT monocularly. The experimental protocol was approved by the Human Ethics Committee of Xi’an Jiaotong University, conforming to the Declaration of Helsinki. All subjects also submitted written consent after being informed about the contents of the experiment.

### Experimental Equipment

Electroencephalography was recorded by a research-grade EEG system (g.USBamp and g.GAMMAbox, g.tec, Schiedlberg, Austria) with a sampling frequency of 1,200 Hz. According to previous studies (Hemptinne et al., 2018; Zheng et al., 2020a), six occipital electrodes (O1, Oz, O2, PO3, POz, and PO4) were used to collect EEG signals. The ground electrode was placed on the forehead (Fpz), and the reference electrode was placed on the left earlobe (A1). In addition, a notch filter from 48 to 52 Hz was applied to eliminate the power line interference. A 24.5-inch LCD monitor (PG258Q, ASUS, Taipei, China) with a resolution of 1,920 × 1,080 pixels and a refresh rate of 240 Hz was used to present visual stimuli.

### Visual Stimuli

**FIGURE 1:**
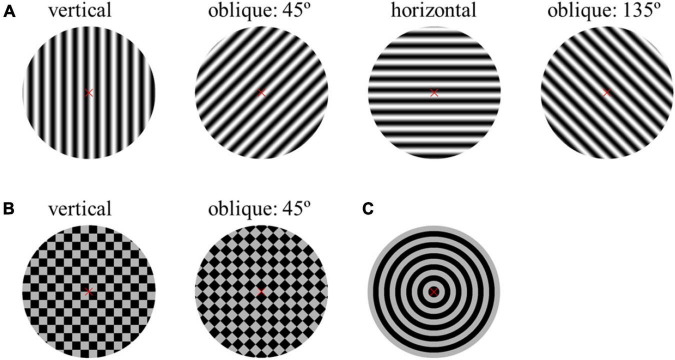
Examples of visual stimuli at the spatial frequency of 3 cpd. **(A)** Reversal sinusoidal gratings with four orientations: vertical, 45°, horizontal, and 135°. **(B)** Reversal checkerboards with two orientations: vertical and 45°. **(C)** Oscillating expansion-contraction concentric-rings. The oblique angle refers to the angle of clockwise rotation against the vertical orientation.

As shown in Figure 1, three types of visual stimuli (i.e., reversal sinusoidal gratings, reversal checkerboards, and oscillating expansion-contraction concentric-rings) with various orientations were used as seven separate experimental runs. Reversal sinusoidal gratings contained four orientations (i.e., vertical, 45°, horizontal, and 135°) corresponding to run A, B, C, and D. Reversal checkerboards contained two orientations (vertical and 45°) corresponding to run E and F. Run G was the visual stimulus of oscillating expansion-contraction concentric-rings. The design details of these visual stimuli were introduced in the previous study (Zheng et al., 2019, 2020a). The visual stimuli were developed by MATLAB (MathWorks, Natick, MA, United States) using the Psychophysics Toolbox (Brainard, 1997).

Six logarithmically equidistant spatial frequency steps of 3.0, 4.8, 7.5, 12.0, 19.0, and 30.0 cycles per degree (cpd) corresponding to the visual acuity optotypes of 1.0, 0.8, 0.6, 0.4, 0.2, and 0.0 logMAR (log minimum angle of resolution) were presented to subjects in each run, among which the dominant spatial frequency at the diagonal orientation of the checkerboards was taken into consideration (Zheng et al., 2020a). The temporal reversal frequency of the visual stimuli was 7.5 Hz (Kurtenbach et al., 2013). The Michelson contrast of the visual stimuli was 50%, and the mean background luminance was 80 cd/m^2^. Besides, a small red cross was presented at the center of the visual stimuli to aid fixation (Almoqbel et al., 2011).

### Experimental Procedure

The distance between the display and subjects was adjusted to ensure the visual angle of the visual stimuli was four degrees, following the recommended parameter settings of previous studies (Almoqbel et al., 2011). Subjects were required to complete the seven runs mentioned above, and each run contained six blocks corresponding to six spatial frequency steps. Each block contained five trials, and each trial lasted 5 s with a 2-s interval between two trials. The order of the seven runs was random, and subjects had enough time to relax between two runs. The experiment was carried out monocularly. In addition, three subjects accomplished both two eyes’ experiments while the others only accomplished one eye’s experiment.

### Signal Processing

#### Data Pre-processing

The start and end times of each trial were used to extract the SSVEP data segments. Then, a band-pass filter from 3 to 40 Hz was imposed to eliminate the high-frequency interferences and low-frequency drifts. The five data segments of the same spatial frequency in one block were averaged to a 5-s data epoch for further data processing.

#### Spatial Filtering

Common average reference (CAR), one of the widely used spatial filtering methods in SSVEPs (Wong et al., 2020), is used to enhance the signal quality. Here, by subtracting the mean of all six electrode signals from the Oz electrode signal, the 5-s single-channel SSVEP signals *V*_*i*_ to be further processed for each epoch can be expressed as follows:


(1) $$V_{i}=V_{Oz}-\frac{1}{6}\sum_{j=1}^{6}V_{j}$$

where *V*_*j*_ corresponds to the SSVEP signal from six record electrodes, respectively.

#### Signal-to-Noise-Ratio

The noise was defined by the mean value of the 20 adjacent amplitudes of either side of the target frequency of 7.5 Hz on the frequency-domain spectrum (Bach and Meigen, 1999; Zheng et al., 2020b). Hence, the signal-to-noise-ratio (SNR) can be determined by the ratio of SSVEP amplitude at 7.5 Hz to noise:


(2) $$SNR=\frac{\mathrm{SSVEP}\mathrm{amplitude}}{\text{noise}}=\frac{a\left(f\right)}{\frac{1}{10}*\sum_{k=1}^{k=10}a\left(f+k*△f\right)+a\left(f-k*△f\right)}$$

where *a(f)* represents the amplitude on the frequency-domain spectrum at target frequency *f* of 7.5 Hz, and frequency resolution Δ*f* is 0.1 Hz.

### Visual Acuity Determination Criterion

For each visual stimulus, SSVEP amplitude can be plotted versus spatial frequency, and then a regression line can be extrapolated from the last significant SSVEP peak to 0 μV. The SSVEP visual acuity was determined as the spatial frequency corresponding to the intersection point with the X-axis, i.e., 0 μV baseline (Zheng et al., 2020b; Hamilton et al., 2021a). The range for the regression line was from the last significant SSVEP peak with an SNR ≥ 3 to the penultimate data point with an SNR ≥ 1 (Hamilton et al., 2021b).

### Statistical Analysis

Statistical analyses were performed by using SPSS 19.0 (IBM, Armonk, NY, United States). Two-way repeated-measures analysis of variance (ANOVA) was introduced to evaluate the significance of the oblique effect on the SSVEP amplitude and SNR of seven visual stimuli of various orientations at six spatial frequencies (Muller and Barton, 1989). The *post hoc* analysis with Bonferroni correction for multiple comparisons was also employed when necessary. One-way repeated-measures ANOVA was also employed to evaluate the difference among the SSVEP visual acuity results obtained by these seven visual stimuli and FrACT visual acuity.

## Results

### SSVEP Response for Each Type of Visual Stimulus

#### Reversal Sinusoidal Gratings

**FIGURE 2:**
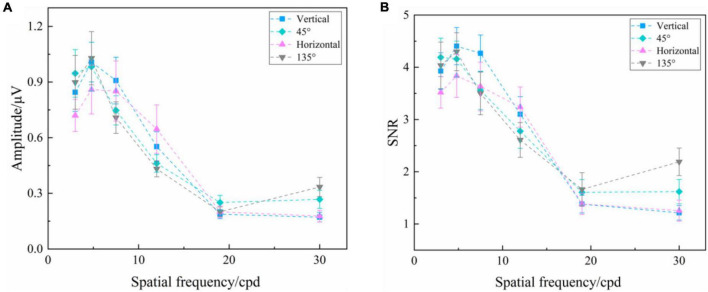
Mean values and SE of SSVEP amplitude and SNR induced by the reversal sinusoidal gratings of vertical, 45°, horizontal, and 135° orientations at six spatial frequency steps over all subjects. **(A)** Amplitude. **(B)** SNR.

As shown in Figure 2, two-way repeated-measures ANOVA found that the interaction of two factors of “stimulus orientation” and “spatial frequency” was significant both in SSVEP amplitude [*F*_(15, 180)_ = 2.540, *P* = 0.002] and SNR [*F*_(15, 180)_ = 2.504, *P* = 0.002] for the reversal sinusoidal gratings. In general, at the same orientation, the high spatial frequency corresponded to the low SSVEP amplitude and SNR, and vice versa, which is also the theoretical basis of SSVEP visual acuity assessment (Hamilton et al., 2021a,b).

One-way repeated-measures ANOVA was also utilized to analyze the difference in amplitude of SSVEPs induced by the reversal sinusoidal gratings of vertical, 45°, horizontal, and 135° orientations at each spatial frequency step, finding a significant difference at the spatial frequency of 3.0 cpd [*F*_(3, 36)_ = 4.099, *P* = 0.013] and 30.0 cpd [Greenhouse–Geisser *F*_(1.825, 21.902)_ = 5.752, *P* = 0.011], but no difference at 4.8 cpd [Greenhouse–Geisser *F*_(1.612, 19.338)_ = 2.951, *P* = 0.085], 7.5 cpd [*F*_(3, 36)_ = 1.108, *P* = 0.358], 12.0 cpd [Greenhouse–Geisser *F*_(1.839_, _22.066)_ = 1.594, *P* = 0.226], and 19.0 cpd [*F*_(3, 36)_ = 1.031, *P* = 0.390]. The subsequent Bonferroni post-hoc analysis of SSVEP amplitude of the reversal sinusoidal gratings of four orientations at the spatial frequency of 3.0 and 30.0 cpd was shown in Supplementary Tables 1, 2, showing that there was no significant difference in SSVEP amplitude among each orientation at 3.0 cpd when the visual stimuli was clear enough to the subjects (*P* > 0.05, respectively), indicating that the stimulus orientation or oblique effect had no significant effect on the amplitude of SSVEPs induced by the reversal sinusoidal gratings. The SNR also had a similar performance.

#### Reversal Checkerboards

**FIGURE 3:**
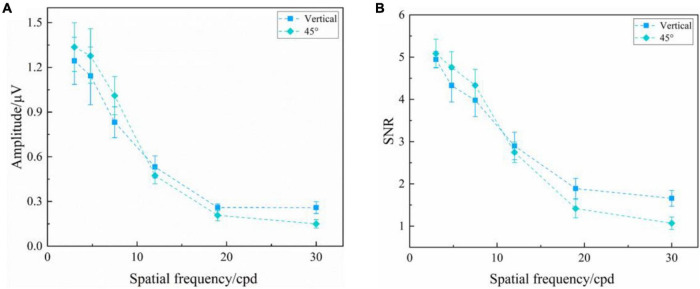
Mean values and SE of SSVEP amplitude and SNR induced by the reversal checkerboards of vertical and 45° orientations at six spatial frequency steps over all subjects. **(A)** Amplitude. **(B)** SNR.

Figure 3 presents the SSVEP amplitude and SNR induced by the reversal checkerboards of vertical and 45° orientations at six spatial frequency steps over all subjects. Two-way repeated-measures ANOVA found that the interaction of two factors of “stimulus orientation” and “spatial frequency” was significant both in SSVEP amplitude [*F*_(5, 60)_ = 2.926, *P* = 0.020] and SNR [*F*_(5_, _60)_ = 2.440, *P* = 0.044] for the reversal checkerboards. In general, at the same orientation, the high spatial frequency corresponded to the low SSVEP amplitude and SNR, and vice versa.

One-way repeated-measures ANOVA was also utilized to analyze the difference in amplitude of SSVEPs induced by the reversal sinusoidal gratings of vertical and 45° orientations at each spatial frequency step, only finding a significant difference at the spatial frequency of 30.0 cpd [*F*_(1, 36)_ = 9.792, *P* = 0.009], but no difference at 3.0 cpd [*F*_(1, 12)_ = 1.798, *P* = 0.205], 4.8 cpd [*F*_(1, 12)_ = 2.590, *P* = 0.134], 7.5 cpd [*F*_(1, 12)_ = 4.019, *P* = 0.068], 12.0 cpd [*F*_(1, 12)_ = 0.543, *P* = 0.476], and 19.0 cpd [*F*_(1, 12)_ = 2.220, *P* = 0.162], indicating that the stimulus orientation or oblique effect had no significant effect on the amplitude of SSVEPs induced by reversal checkerboards. The SNR also had a similar performance.

#### Oscillating Expansion-Contraction Concentric-Rings

**FIGURE 4:**
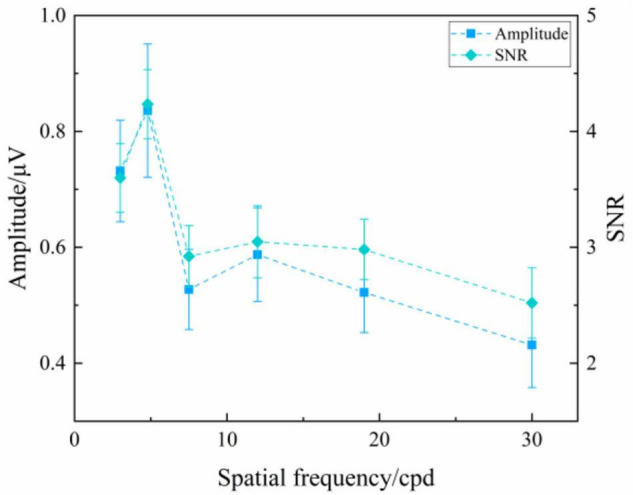
Mean values and SE of SSVEP amplitude and SNR induced by the oscillating expansion-contraction concentric-rings at six spatial frequency steps over all subjects.

Figure 4 presents the SSVEP amplitude and SNR induced by the oscillating expansion-contraction concentric-rings at six spatial frequency steps over all subjects. The SSVEP amplitude and SNR changed with the spatial frequency changing. In general, the high spatial frequency corresponded to the low SSVEP amplitude, and vice versa, which was similar to the reversal sinusoidal gratings and the reversal checkerboards. As shown in Figure 4, the reason for the small notch both in SSVEP amplitude and SNR at the intermediate spatial frequencies, i.e., 7.5 cpd, could be because the SSVEPs from two of all subjects had a lower response at this spatial frequency step, which occasionally occurred in SSVEP visual acuity study (Bach et al., 2008; Ridder et al., 2012).

### Comparison of SSVEP Response of Three Types of Visual Stimuli

**FIGURE 5:**
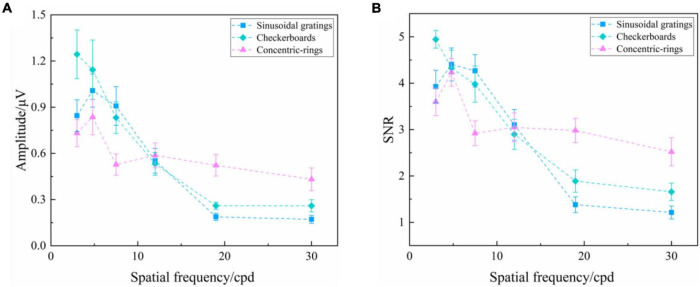
Mean values and SE of SSVEP amplitude and SNR induced by the reversal sinusoidal gratings of vertical orientation, reversal checkerboards of vertical orientation, and oscillating expansion-contraction concentric-rings at six spatial frequency steps over all subjects. **(A)** Amplitude. **(B)** SNR.

As analyzed above, the orientation of visual stimuli did not affect the SSVEP response. Hence, we chose SSVEPs induced by reversal sinusoidal gratings of vertical orientation, reversal checkerboards of vertical orientation, and oscillating expansion-contraction concentric-rings all at 3.0 cpd to compare the characteristics of SSVEPs induced by these three types of visual stimuli. To explore the effect of stimulus type on the SSVEP response of various spatial frequency steps, as shown in Figure 5, two-way repeated-measures ANOVA found that the interaction of two factors of “stimulus type” and “spatial frequency” was significant in SSVEP amplitude [Greenhouse–Geisser *F*_(3.369_, _40.430)_ = 9.479, *P* < 0.001] and SNR [*F*_(10_, _120)_ = 7.894, *P* < 0.001].

Subsequently, the difference in SSVEP amplitude of the three types of visual stimuli at each spatial frequency step was analyzed by one-way repeated-measures ANOVA, finding a significant difference among three types of visual stimuli at the spatial frequency of 3.0 cpd [*F*_(2, 24)_ = 11.488, *P* < 0.001], 7.5 cpd [*F*_(2, 24)_ = 4.059, *P* = 0.030], 19.0 cpd [Greenhouse–Geisser *F*_(1,264, 15.171)_ = 13.838, *P* = 0.001], and 30.0 cpd [Greenhouse–Geisser *F*_(1,294, 15.522)_ = 7.803, *P* = 0.009], but no difference at 4.8 cpd [*F*_(2, 24)_ = 3.251, *P* = 0.056] and 12.0 cpd [*F*_(2, 24)_ = 0.154, *P* = 0.858]. The subsequent Bonferroni *post hoc* analysis of the SSVEP amplitude of three types of visual stimuli at the spatial frequency of 3.0, 7.5, 19.0, and 30.0 cpd was shown in Supplementary Tables 3–6, respectively. The SNR had a similar performance.

Both the SSVEP amplitude and SNR indicated a difference in SSVEP response among these three types of visual stimuli, with the reversal checkerboards showing the largest evoked intensity and the oscillating expansion-contraction concentric-rings showing the smallest evoked intensity at the lower spatial frequencies, such as 3.0 and 7.5 cpd. However, at the higher spatial frequencies, such as 19.0 and 30.0 cpd, the oscillating expansion-contraction concentric-rings showed a larger evoked intensity than the other two types of visual stimuli, indicating that the evoked intensity of oscillating expansion-contraction concentric-rings had a slower downtrend against increasing spatial frequency than that of the reversal sinusoidal gratings and the reversal checkerboards.

### Comparison of Visual Acuity Results

**FIGURE 6:**
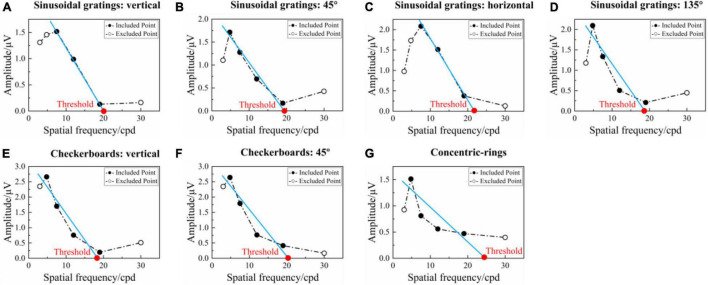
Example of the tuning curves corresponding to the seven runs for SSVEP visual acuity estimation criterion (left eye, subject S4). **(A–D)** Reversal sinusoidal gratings of vertical, 45°, horizontal, and 135° orientations, respectively. **(E,F)** Reversal checkerboards of vertical and 45° orientation. **(G)** Oscillating expansion-contraction concentric-rings. In each subfigure, the blue solid line represents the regression line between the SSVEP amplitude and spatial frequency extrapolated from the last significant SSVEP peak to the penultimate point. The red point is the intersection of the regression line and 0 μV, with its corresponding spatial frequency value defined as the visual acuity threshold.

Steady-state visual evoked potential visual acuity can be defined by extrapolating a straight line regressed through significant SSVEP amplitudes versus spatial frequency to a 0 μV baseline (Zheng et al., 2020b). Figure 6 shows an example of tuning curves corresponding to the seven runs for the SSVEP visual acuity estimation criterion. As for the reversal sinusoidal gratings of vertical orientation in Figure 6A, there was a regression line between the third and fifth points, and the SSVEP visual acuity can be determined as the spatial frequency of the intersection point of the regression line and 0 μV, i.e., 20.106 cpd. Similar to this, the visual acuities for Figures 6B–G were 19.802, 21.672, 18.929, 18.819, 20.037, and 24.829 cpd, respectively. Since the uniformity in spatial frequency (Bach, 2007), the unit of logMAR was used in the final visual acuity expression. Hence, after the conversion to the unit of logMAR, the visual acuities for Figures 6A–G were 0.174, 0.180, 0.141, 0.200, 0.203, 0.175, and 0.082 logMAR, respectively.

**FIGURE 7:**
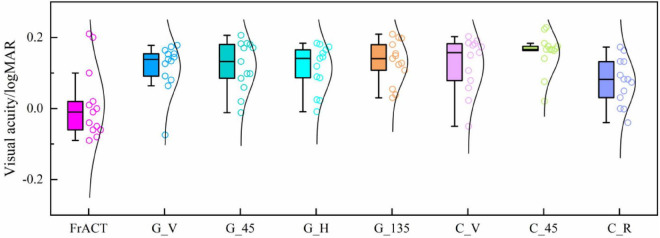
Comparison of the visual acuity assessed by FrACT and SSVEPs of seven visual stimuli over all subjects. G_V, G_45, G_H, and G_135 represent the reversal sinusoidal gratings of vertical, 45°, horizontal, and 135° orientations. C_V and C_45 represent the reversal checkerboards of vertical and 45° orientation. C_R represents the oscillating expansion-contraction concentric-rings.

Figure 7 and Table 1 show the visual acuity estimated by eight tests, i.e., the subjective FrACT test and the objective SSVEPs of seven various visual stimuli, over all subjects. One-way repeated-measures ANOVA found a significant difference in visual acuity among these eight tests [*F*_(7, 84)_ = 3.848, *P* = 0.001]. Then, Bonferroni *post hoc* analysis, as shown in Supplementary Table 7, indicates no difference in the visual acuity among these methods (*P* > 0.05, respectively), demonstrating that the SSVEPs induced by all seven visual stimuli had a similarly good performance in evaluating visual acuity and the stimulus orientation had little effect on the SSVEP visual acuity.

**TABLE 1:** Visual acuity assessed by FrACT and SSVEPs of seven visual stimuli over all subjects.

Subject No.	Eye	FrACT/logMAR	SSVEP/logMAR						
			G_V	G_45	G_H	G_135	C_V	C_45	C_R
S1	Left	–0.09	0.16	0.18	0.18	0.18	0.18	0.18	0.17
S2	Right	0.01	0.15	0.17	0.09	0.15	0.06	0.17	0.09
S3	Left	0.02	0.13	0.18	0.18	0.05	0.15	0.22	0.08
	Right	–0.06	0.09	0.09	0.12	0.14	0.05	0.14	0.03
S4	Left	–0.08	0.17	0.18	0.14	0.20	0.20	0.18	0.08
	Right	–0.04	0.14	0.17	0.16	0.20	0.18	0.23	0.00
S5	Right	0.00	0.15	0.21	0.15	0.21	0.18	0.17	0.13
S6	Left	0.21	0.06	0.06	0.17	0.15	0.19	0.17	0.00
	Right	–0.01	0.13	0.02	0.01	0.03	0.11	0.16	0.05
S7	Right	–0.06	0.14	0.13	0.17	0.12	0.16	0.17	0.16
S8	Right	0.20	0.18	0.01	0.03	0.04	0.08	0.02	0.07
S9	Left	0.10	0.07	0.10	0.02	0.13	0.17	0.18	0.13
S10	Left	–0.05	0.08	0.10	0.09	0.11	0.02	0.08	0.04
Average		0.01	0.11	0.12	0.11	0.13	0.13	0.16	0.07
SD		0.10	0.06	0.07	0.06	0.06	0.07	0.05	0.06

## Discussion

In this study, we aimed to explore that whether there was an effect on SSVEP visual acuity from the oblique effect. We compared the SSVEPs induced by the sinusoidal gratings with horizontal, two oblique, and vertical orientations, checkerboards with vertical and oblique orientation, and oscillating expansion-contraction concentric-rings at six spatial frequency steps. Taking the SSVEP amplitude and SNR of the fundamental frequency as signal characteristics, this study found that both the SSVEP amplitude and SNR induced by the reversal sinusoidal gratings at 3.0 cpd of four stimulus orientations had no difference. The same finding was also shown in the checkerboards with vertical and oblique orientation. By using the visual acuity determination estimation criterion, the SSVEP visual acuity for each visual stimulus was obtained, finding that there was no difference in the visual acuity among these methods. In general, this study demonstrated that the SSVEPs induced by all these seven visual stimuli had a similarly good performance in evaluating visual acuity, and the oblique effect or the stimulus orientation had little effect on the SSVEP response as well as the SSVEP visual acuity.

Scalp-recorded SSVEPs rely on the activation of a large number of neurons, which is mainly concentrated in the primary visual cortex, i.e., V1 region (Norcia et al., 2015). Although some previous studies have mentioned that the orientation effect mainly emanates from the primary visual cortex since fewer neurons optimally respond to the oblique objects at the central part of the retina (Furmanski and Engel, 2000; Li and Gilbert, 2002; Li et al., 2003), evidence for this is weak and controversial (Freeman et al., 2011). Recently, some studies indicated that the selectivity for horizontal and vertical orientations lies in higher-level visual areas (Koelewijn et al., 2011), e.g., parahippocampal place area (PPA) (Epstein and Kanwisher, 1998; Nasr and Tootell, 2012), causing the oblique effect. Our results also supported the opinion that the oblique effect in visual perception is mainly contributed by the higher visual areas rather than the primary visual cortex since the orientation of the visual stimuli had little effect on SSVEP response. In addition, some differences in SSVEP response also occurred among various stimulus orientations at other spatial frequency steps, such as the SSVEP amplitude and SNR induced by the reversal sinusoidal gratings of four orientations at each spatial frequency step of 30.0 and 4.8 cpd, as shown in Figure 2. This may be caused by the signal fluctuation being influenced by the external environment and subjective mental state especially when the stimulus parameter is close to the threshold at a high spatial frequency (Zheng et al., 2019), the notch occasionally occurring at the intermediate spatial frequencies in some subjects (Bach et al., 2008), or the slight difference of the oblique effect at the various spatial frequencies (Nelson et al., 1984).

As for the visual stimuli used in this study, the sinusoidal gratings and checkerboards are the most used stimulus patterns in SSVEP visual acuity assessment (Zheng et al., 2020c; Hamilton et al., 2021b). Based on the vertical orientation, the sinusoidal gratings can form four different types of vertical, 45°, horizontal, and 135° orientations by rotating clockwise per 45°. Similarly, checkerboards can also form two different types of vertical and 45° orientations, since the types of horizontal and 135° orientations are the same as the vertical and 45° orientations, respectively. The motion stimulus pattern of oscillating expansion-contraction concentric-rings was proposed in the previous study (Zheng et al., 2019), and some comparisons have been done to find that it also can be a stimulus paradigm in SSVEP visual acuity assessment with good performance and even some superiorities (Zheng et al., 2020a), e.g., a relatively superior anti-fatigue efficacy (Zheng et al., 2020d). As for the effect of orientation, no matter how you rotate them, the concentric-rings coincide. Hence, this pattern has only one orientation, which can well avoid the possible potential influence of stimulus orientation on SSVEP visual acuity results. Besides, another sometimes used stimulus pattern is the square-wave gratings. Since the characteristics of square-wave gratings are very similar to sinusoidal gratings (Zheng et al., 2020a,d), we only used the pattern of sinusoidal gratings as a representative here.

As for the SSVEP response among three types of visual stimuli, since the V1 region may prefer one-dimensional stripes, the SSVEP response of concentric-rings was lower than sinusoidal gratings and checkerboards at low spatial frequency. Whereas, at the high spatial frequency, the SSVEP response of concentric-rings was higher than sinusoidal gratings and checkerboards, which may be caused by the motion style and anti-fatigue characteristics of steady-state motion visual evoked potentials (SSMVEPs) (Zheng et al., 2019, 2020d). Besides, as checkerboards can be regarded as the intersection of vertical stripes and horizontal stripes, the SSVEP response of checkerboards was always higher than sinusoidal gratings.

In addition, though the 7.5 Hz temporal frequency was recommended in SSVEP visual acuity, there may be also some difference of oblique effect on various temporal frequencies (Nelson et al., 1984). Hence, further work is required to estimate whether the oblique effect can cause some difference in SSVEPs with various temporal frequencies. Besides, to explore the common effect of oblique effect on SSVEPs in various spatial frequencies, in this study, the visual acuity of the subjects was normal or corrected to normal. Further research could also be conducted to explore the difference of oblique effect on SSVEP acuity in subjects with lower visual acuity. Finally, since some effect also occurred on SSVEP response among various stimulus orientations at some spatial frequency steps as mentioned above, we recommended using a consistent visual stimulus paradigm and orientation during SSVEP visual acuity, minimizing the impact on the final results.

## Conclusion

This study compared the SSVEPs induced by seven visual stimuli of the sinusoidal gratings with horizontal, two oblique, and vertical orientations, checkerboards with vertical and oblique orientation, and oscillating expansion-contraction concentric-rings at six spatial frequency steps. The results showed that both the SSVEP amplitude and SNR induced by the reversal sinusoidal gratings at 3.0 cpd of four stimulus orientations had no difference, and the same finding was also shown in the checkerboards with vertical and oblique orientation, showing that stimulus orientation of the reversal sinusoidal gratings and checkerboards had no significant effect on the SSVEP response. The SSVEP visual acuity obtained by the threshold determination estimation criterion for all seven visual stimuli also showed no difference. This study demonstrated that the SSVEPs induced by all these seven visual stimuli had a similarly, good performance in evaluating visual acuity, and the oblique effect or the stimulus orientation had little effect on SSVEP response as well as the SSVEP visual acuity.

## Data Availability Statement

The raw data supporting the conclusions of this article will be made available by the authors, without undue reservation.

## Ethics Statement

The studies involving human participants were reviewed and approved by Human Ethics Committee of Xi’an Jiaotong University. The patients/participants provided their written informed consent to participate in this study.

## Author Contributions

XZ contributed to the study design, data acquisition, analysis, interpretation, and manuscript writing, and revision. GX contributed to the study design and approval of the final version for publication. YD and HL contributed to the statistical analysis and manuscript drafting. CH and PT contributed to the data analysis and interpretation. ZL and CD contributed to the manuscript writing and revision. WY provided the experimental equipment and approved the final version for publication. SZ conceptualized the study. All authors contributed to the article and approved the submitted version.

## Conflict of Interest

The authors declare that the research was conducted in the absence of any commercial or financial relationships that could be construed as a potential conflict of interest.

## Publisher’s Note

All claims expressed in this article are solely those of the authors and do not necessarily represent those of their affiliated organizations, or those of the publisher, the editors and the reviewers. Any product that may be evaluated in this article, or claim that may be made by its manufacturer, is not guaranteed or endorsed by the publisher.
