# Supplementary material for: Does Oblique Effect Affect SSVEP-Based Visual Acuity Assessment?
Source: Front Neurosci. 2022 Jan 14;15:784888. doi: 10.3389/fnins.2021.784888 (PMC8795862; doi:10.3389/fnins.2021.784888)
Supplement: Supplementary file 1 [file Table_1.DOCX]

Table 1. Bonferroni *post-hoc* analysis of SSVEP amplitude induced by the reversal sinusoidal gratings of vertical, 45º, horizontal, and 135º orientations at the spatial frequency of 3 cpd.

| **Orientation** | **45º** | **Horizontal** | **135º** |
| --- | --- | --- | --- |
| **Vertical** | *P* = 0.959 | *P* = 0.303 | *P* = 1.000 |
| **45º** | ― | *P* = 0.083 | *P* = 1.000 |
| **Horizontal** | ― | ― | *P* = 0.215 |

Table 2. Bonferroni *post-hoc* analysis of SSVEP amplitude induced by the reversal sinusoidal gratings of vertical, 45º, horizontal, and 135º orientations at the spatial frequency of 30.0 cpd. **P* < 0.05.

| **Orientation** | **45º** | **Horizontal** | **135º** |
| --- | --- | --- | --- |
| **Vertical** | *P* = 0.403 | *P* = 1.000 | *P* = 0.042* |
| **45º** | ― | *P* = 0.886 | *P* = 0.387 |
| **Horizontal** | ― | ― | *P* = 0.050 |

Table 3. Bonferroni *post-hoc* analysis of SSVEP amplitude among three types of visual stimuli at the spatial frequency of 3.0 cpd. ****P* < 0.001; ***P* < 0.01; **P* < 0.05.

| **Type** | **Checkerboards** | **Concentric-rings** |
| --- | --- | --- |
| **Gratings** | *P* = 0.010* | *P* = 0.732 |
| **Checkerboards** | ― | *P* = 0.006** |

Table 4. Bonferroni *post-hoc* analysis of SSVEP amplitude among three types of visual stimuli at the spatial frequency of 7.5 cpd.

| **Type** | **Checkerboards** | **Concentric-rings** |
| --- | --- | --- |
| **Gratings** | *P* = 1.000 | *P* = 0.100 |
| **Checkerboards** | ― | *P* = 0.138 |

Table 5. Bonferroni *post-hoc* analysis of SSVEP amplitude among three types of visual stimuli at the spatial frequency of 19.0 cpd. ****P* < 0.001; ***P* < 0.01; **P* < 0.05.

| **Type** | **Checkerboards** | **Concentric-rings** |
| --- | --- | --- |
| **Gratings** | *P* = 0.147 | *P* = 0.003** |
| **Checkerboards** | ― | *P* = 0.021* |

Table 6. Bonferroni *post-hoc* analysis of SSVEP amplitude among three types of visual stimuli at the spatial frequency of 30.0 cpd. ****P* < 0.001; ***P* < 0.01; **P* < 0.05.

| **Type** | **Checkerboards** | **Concentric-rings** |
| --- | --- | --- |
| **Gratings** | *P* = 0.099 | *P* = 0.011* |
| **Checkerboards** | ― | *P* = 0.182 |

Table 7. Bonferroni *post-hoc* analysis of visual acuity obtained by the subjective FrACT test and the objective SSVEPs of seven various visual stimuli. **P* < 0.05. G_V, G_45, G_H, and G_135 represent the reversal sinusoidal gratings of vertical, 45º, horizontal, and 135º orientations. C_V and C_45 represent the reversal checkerboards of vertical and 45º orientation. C_R represents the oscillating expansion-contraction concentric-rings.

| **Test** | **G_V** | **G_45** | **G_H** | **G_135** | **C_V** | **C_45** | **C_R** |
| --- | --- | --- | --- | --- | --- | --- | --- |
| **FrACT** | *P* = 0.812 | *P* = 1.000 | *P* = 1.000 | *P* = 1.000 | *P* = 0.600 | *P* = 0.331 | *P* = 1.000 |
| **G_V** | ― | *P* = 1.000 | *P* = 1.000 | *P* = 1.000 | *P* = 1.000 | *P* = 1.000 | *P* = 0.214 |
| **G_45** | ― | ― | *P* = 1.000 | *P* = 1.000 | *P* = 1.000 | *P* = 0.979 | *P* = 1.000 |
| **G_H** | ― | ― | ― | *P* = 1.000 | *P* = 1.000 | *P* = 0.530 | *P* = 1.000 |
| **G_135** | ― | ― | ― | ― | *P* = 1.000 | *P* = 1.000 | *P* = 1.000 |
| **C_V** | ― | ― | ― | ― | ― | *P* = 1.000 | *P* = 0.583 |
| **C_45** | ― | ― | ― | ― | ― | ― | *P* = 0.093 |
